# Supplementary material for: Replication of population-level differences in auditory-motor synchronization ability in a Norwegian-speaking population
Source: Commun Psychol. 2023 Dec 20;1:47. doi: 10.1038/s44271-023-00049-2 (PMC11332004; doi:10.1038/s44271-023-00049-2)
Supplement: Supplementary file 2 — Supplementary Information [file 44271_2023_49_MOESM2_ESM.pdf]

Supplementary Materials

Supplementary Figure 1

A.

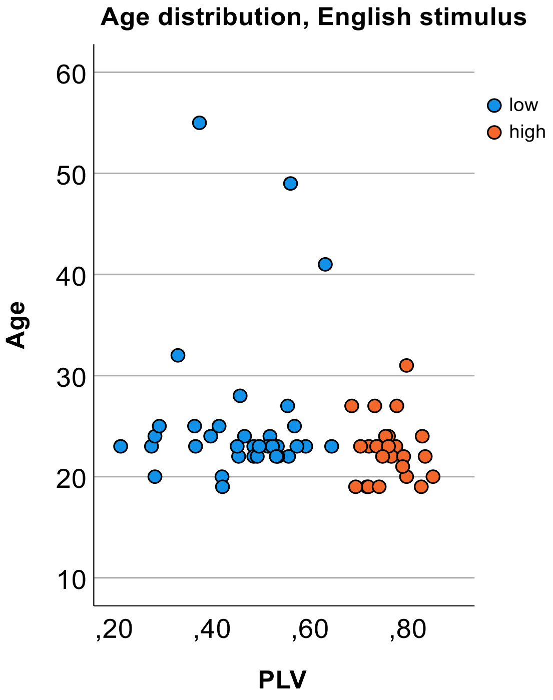

B.

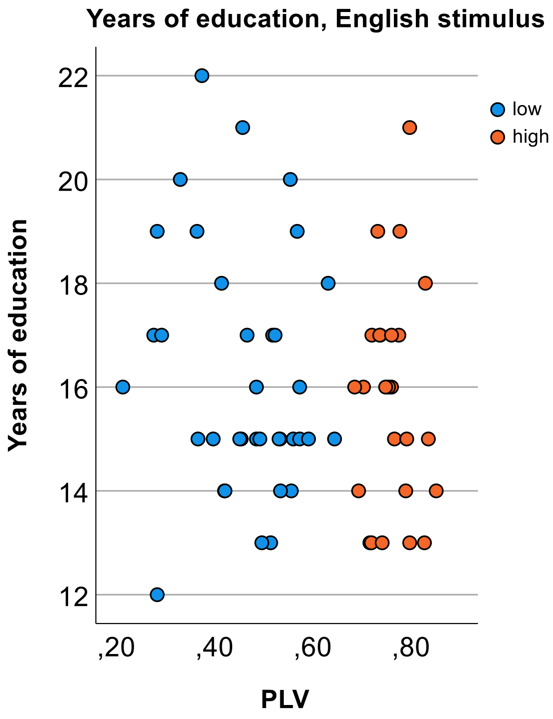

C.

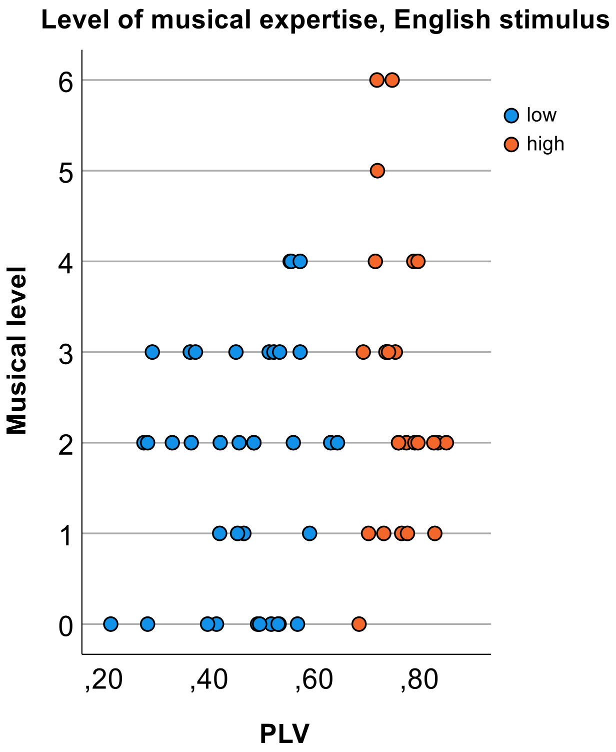

D.

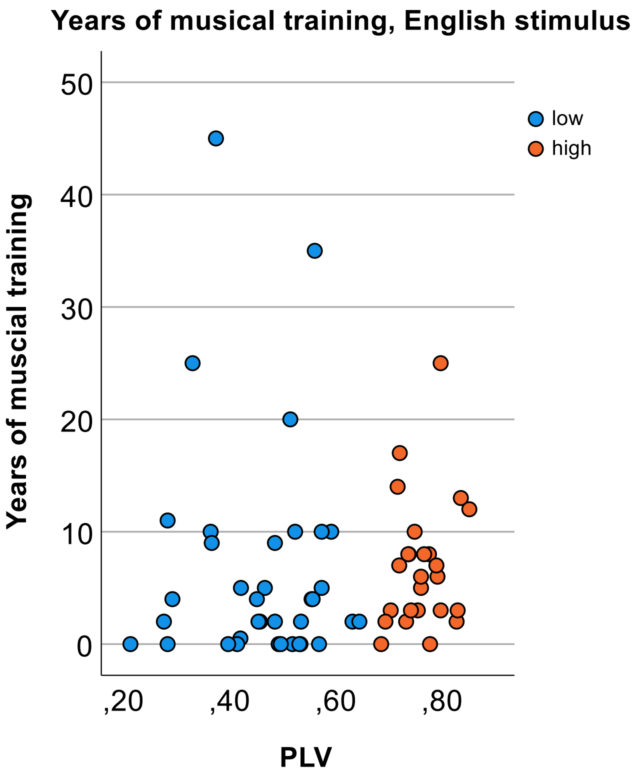

E.

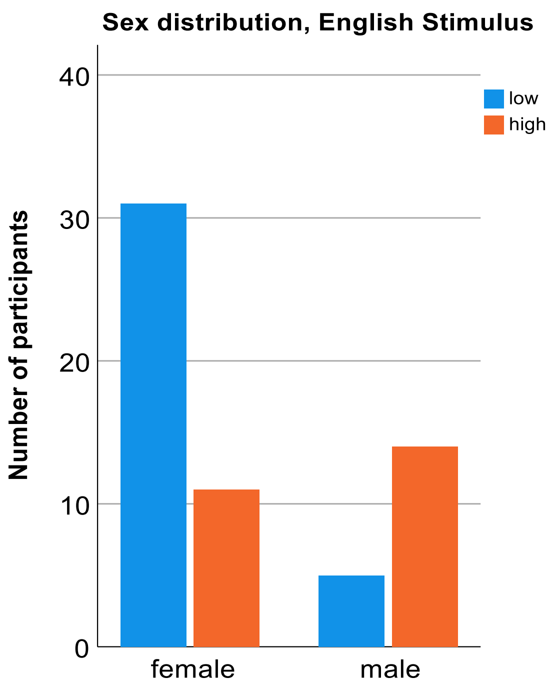
Sex distribution, English Stimulus
Number of participants
40
30
20
10
0
low
high
female
male

***Supplementary Figure 1: Comparisons of low and high synchronizers, English stimulus*. ** n lows = 36; n highs = 25. PLV = Phase-locking value. **A.** Age distribution. Close to significant difference between groups (*lows*: median = 23, standard deviation = 7.6; *highs*: median = 23, standard deviation = 3.0; rank-biserial, r = -.23; Mann-Whitney U test, two-sided, U = 312, *P* = .055, 95% CI [22.73, 25.97]). **B.** Distribution for years of education. No significant difference between groups (*lows*: median = 15, standard deviation = 2.4; *highs*: median = 16, standard deviation = 2.2; rank-biserial correlation, r = -.10; Mann-Whitney U test, two-sided, U = 412.5, *P* = .583, 95% CI [15.45, 16.62]). **C.** Distribution of musical expertise. Close to significant differences between groups (*lows:* mean = 1.7, standard deviation = 1.3, *highs:* mean = 2.6, standard deviation = 1.6; rank-biserial correlation, r = .30; Mann-Whitney U test, two-sided, U = 322.5, *P* = .055, 95% CI [1.68, 2.45]). **D.** Distribution of years of musical training. No significant difference between groups (*lows:* mean = 6.7, standard deviation = 10.2, *highs*: mean = 7.0, standard deviation = 5.8; rank-biserial correlation, r = .02; Mann-Whitney U test, two-sided, U = 342.5, *P* = .114, 95% CI [4.61, 9.04]). 12 participants had no musical training. **E.** Sex distribution. Significant association between group membership and sex (*lows:* female participants n = 31, male participants n = 5, *highs:* female participants n = 11, male participants n = 14; Fischer's exact test, two-sided; *P* <.001; Odds Ratio = 7.89, 95% CI [2.30, 27.03]).

Supplementary Figure 2

A.

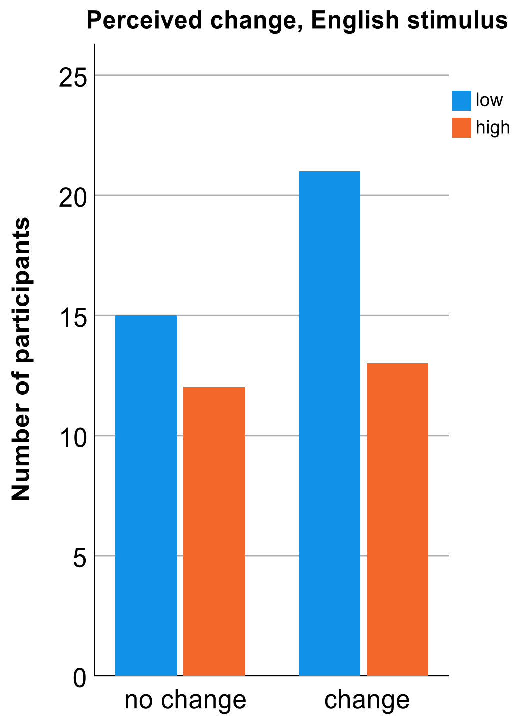

B.

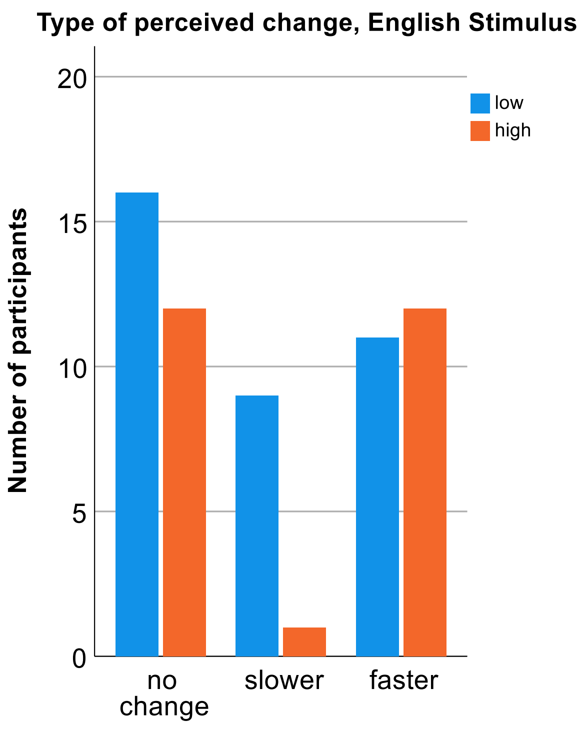

C.

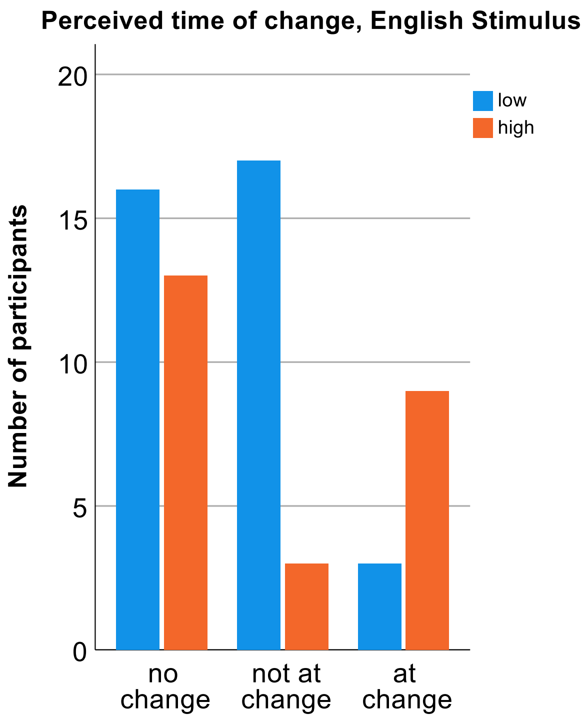

Supplementary Figure 2: Perceived change in stimulus, English stimulus. n lows = 36; n highs = 25. **A.** Whether the participant perceived a change in the stimulus. **B.** Whether the change was perceived as an increment or decrement. **C.** Whether they perceived the exact timing of the change. In total, 13 participants correctly identified the time and nature of the change in the stimulus.

Supplementary Figure 3

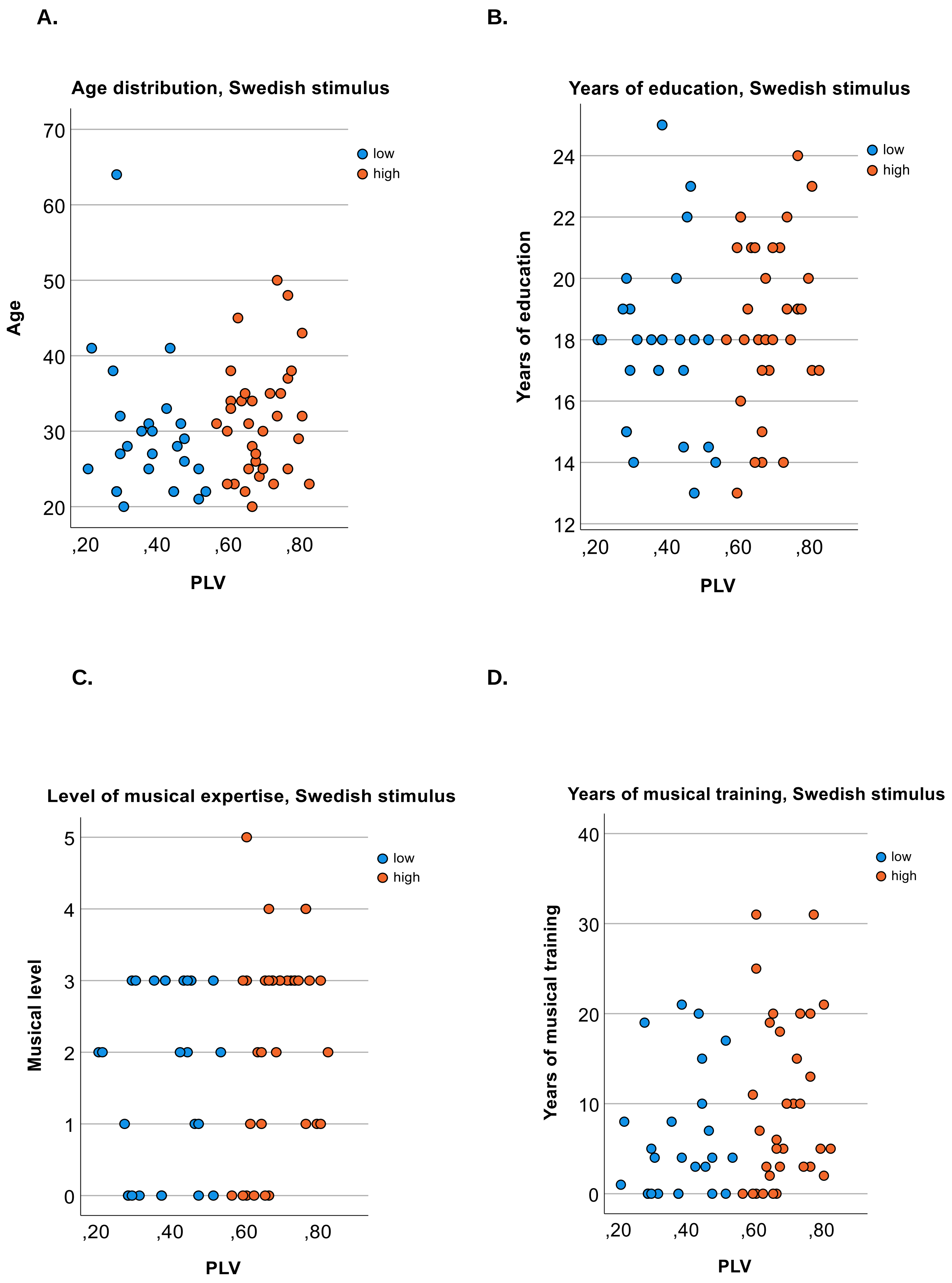

E.

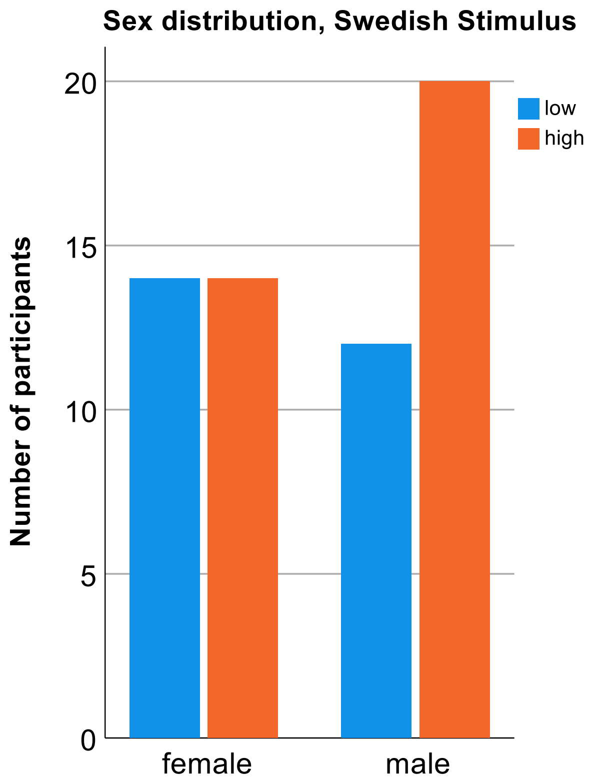
Sex distribution, Swedish Stimulus
Number of participants
20
15
10
5
0
female
male
low
high

***Supplementary Figure 3: Comparisons of low and high synchronizers, Swedish stimulus*.** n lows = 26; n highs = 34. PLV = Phase-locking value.

A. Age distribution. No significant difference between groups (*lows:* median = 27.5, standard deviation = 9.13, *highs:* median = 31, standard deviation = 7.5; rank-biserial correlation, r = .13; Mann-Whitney U test, two-sided, U = 333, *P* = .131, 95% CI [28.37, 32,63]). **B.** Distribution for years of education. No significant difference between groups (*lows:* median = 18, standard deviation = 2.8, *highs:* median = 18.5, standard deviation = 2.7; rank-biserial correlation, r = .17; Mann-Whitney U test, two-sided, U = 344.5, *P* = .143, 95% CI [17.50, 18.94]).**C.** Distribution of musical expertise. Close to significant differences between groups (*lows:* mean = 1.5 standard deviation = 1.3, *highs:* mean = 2.2, standard deviation = 1.4; rank-biserial correlation, r = .24; Mann-Whitney U test, two-sided, U = 320, *P* = .057, 95% CI [1.56, 2.27]). **D.** Distribution of years of musical training. No significant difference between groups (*lows:* mean = 5.8, standard deviation = 6.9, *highs*: mean = 9.8, standard deviation = 9.1; rank-biserial correlation, r = .23; Mann-Whitney U test, two-sided, U = 323.5, *P* = .074, 95% CI [5.93, 10.27]). 15 participants had no musical training. **E.** Sex distribution. No significant association between group membership and sex (*lows*: female participants n = 14, male participants n = 14, *highs*: female participants n = 12, male participants n = 20; Fischer's exact test, two-sided, *P* = .435; Odds Ratio = 1.56, 95% CI [.56, 4.32]).

Supplementary Figure 4

A.

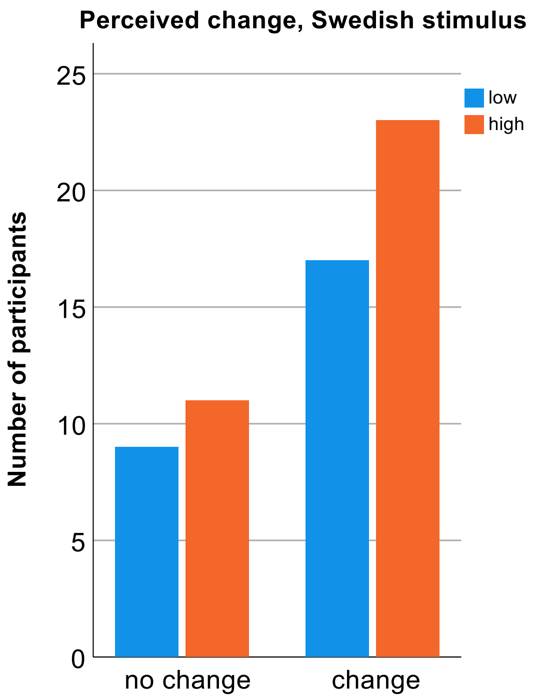

B.

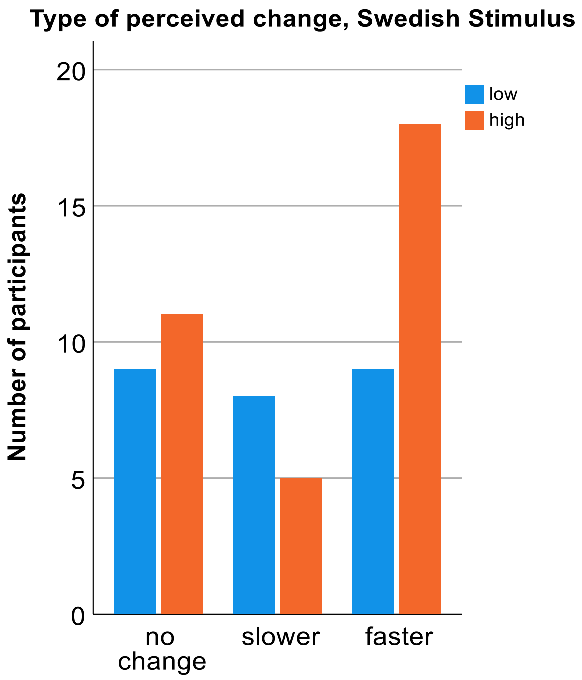

C.

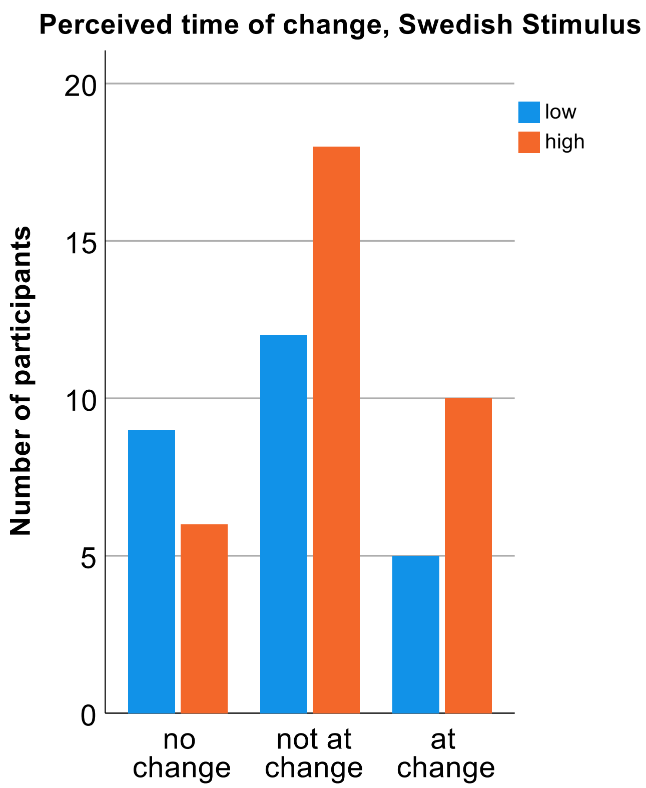

Supplementary Figure 4: Perceived change in stimulus, Swedish stimulus. n lows = 26; n highs = 34). **A.** Whether the participant perceived a change in the stimulus. **B.** Whether the change was perceived as an increment or decrement. **C.** Whether they perceived the exact timing of the change. In total, 14 participants correctly perceived the time and nature of the change.

Supplementary Figure 5.

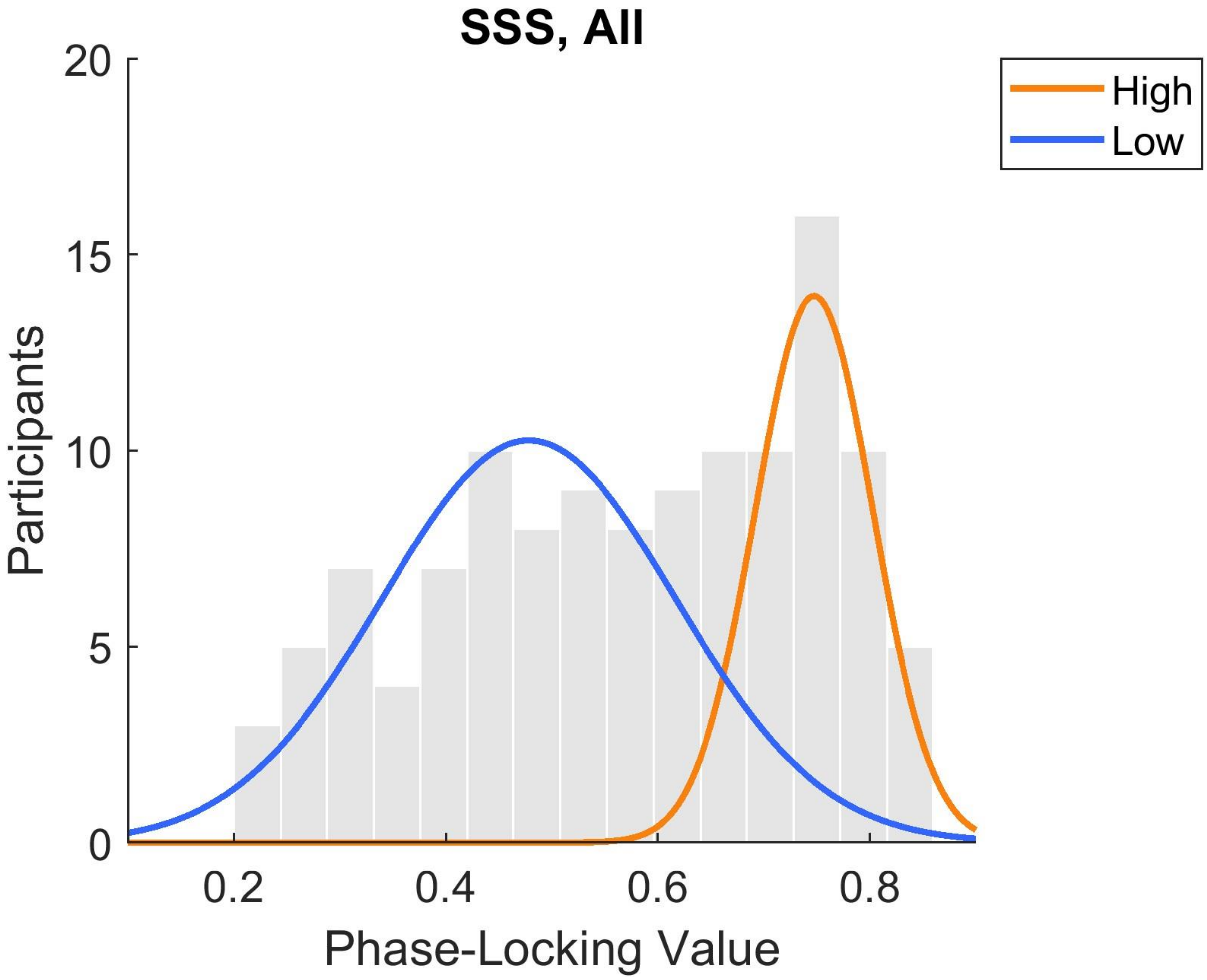

Supplementary Figure 5: Combined distribution. PLVs from the English stimulus cohort and the Swedish stimulus cohort combined. Distributions obtained by fitting the data with a two component Gaussian mixture model. The distribution is non-uniform. N = 121. Low synchronizers (n = 74, component weight coefficient = 0.65, mean = 0.48); High synchronizers (n = 47, component weight coefficient = 0.35, mean = 0.75). One-sample Kolmogorov-Smirnov test, two-sided: D(121) = 0.58, P < .001, CI 95% [.014, .019].

Supplementary Figure 6

A.

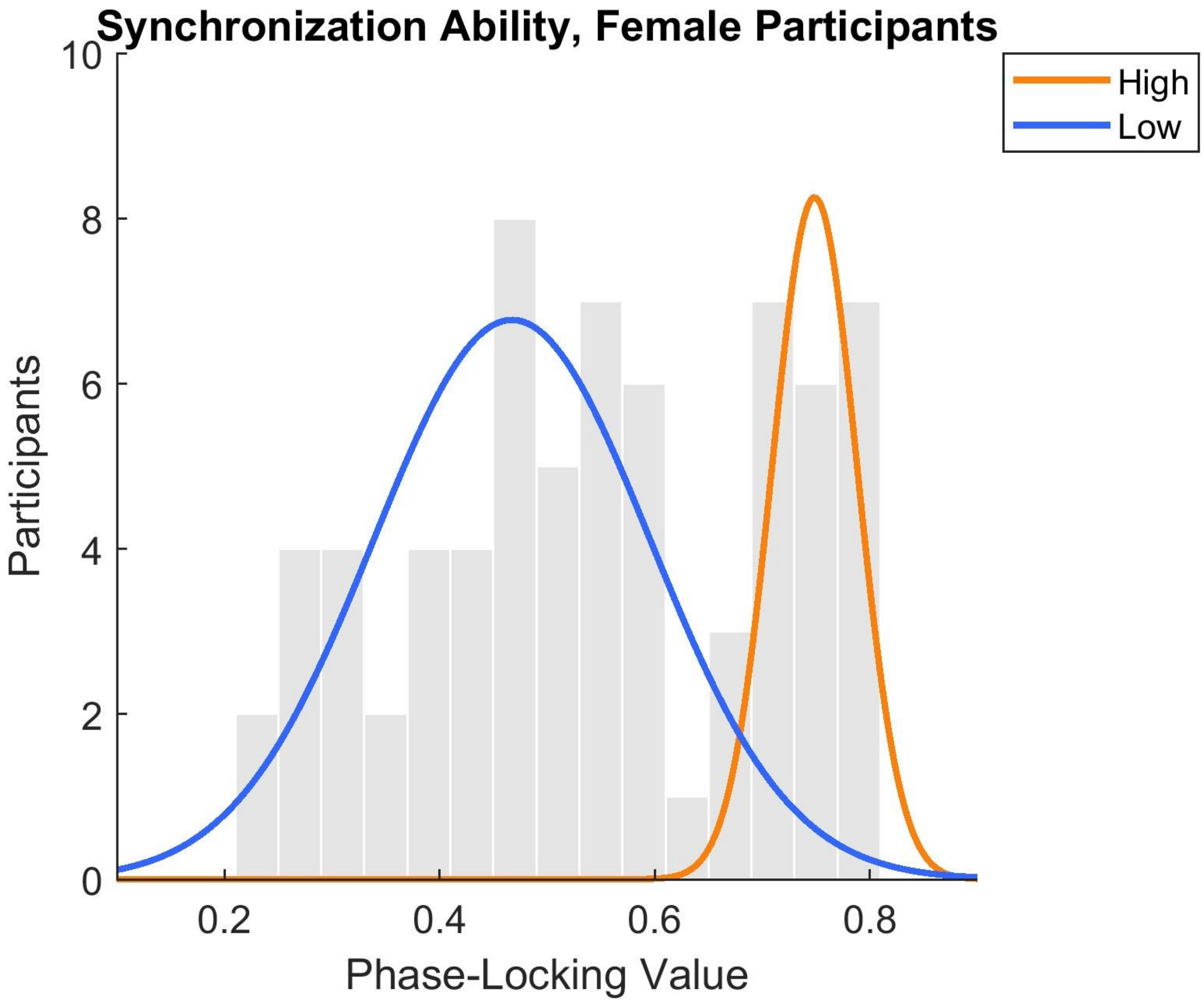

B.

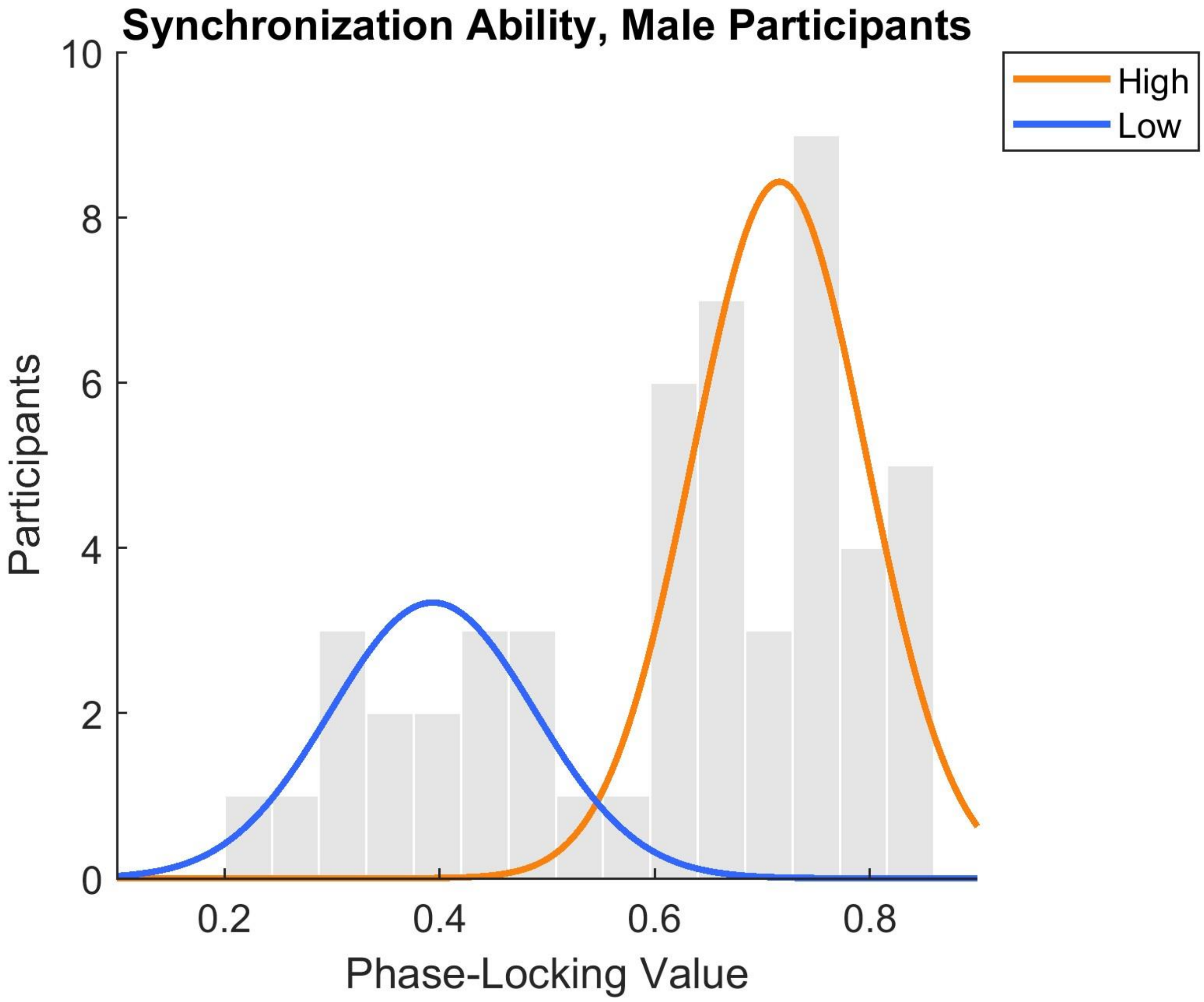

Supplementary Figure 6: Distribution stratified by sex. Given our results of an association between group membership and sex for the English stimulus cohort, but not for the Swedish stimulus cohort, we explored the distribution of PLVs for each sex (across the two cohorts). Distributions obtained by fitting the data with a two component Gaussian mixture model. Both distributions are non-uniform. **A.** Female participants, n = 70. Low synchronizers (n = 49, component weight coefficient = 0.73, mean = 0.47); high synchronizers (n = 21, component weight coefficient = 0.27, mean = 0.75. One-sample Kolmogorov-Smirnov test, two-sided, D(70) = 0.59, P < .001, 95% CI [.062, .072]. **B.** Male participants, n = 51. Low synchronizers (n lows = 16, component weight coefficient = 0.32, mean = 0.39); high synchronizers (n = 35, component weight coefficient = 0.68, mean = 0.72). One-sample Kolmogorov-Smirnov test, two-sided: D(51) = 0.59, P < .001, 95% CI [.014, .019].
