# Supplementary material for: Replication of population-level differences in auditory-motor synchronization ability in a Norwegian-speaking population
Source: Commun Psychol. 2023 Dec 20;1:47. doi: 10.1038/s44271-023-00049-2 (PMC11332004; doi:10.1038/s44271-023-00049-2)
Supplement: Supplementary file 3 — Reporting Summary [file 44271_2023_49_MOESM3_ESM.pdf]

nature portfolio

Corresponding author(s): Guro S. Sjuls

Last updated by author(s): Nov 10, 2023

Reporting Summary

Nature Portfolio wishes to improve the reproducibility of the work that we publish. This form provides structure for consistency and transparency in reporting. For further information on Nature Portfolio policies, see our Editorial Policies and the Editorial Policy Checklist.

Statistics

For all statistical analyses, confirm that the following items are present in the figure legend, table legend, main text, or Methods section.

n/a	Confirmed	
☐	☒	The exact sample size (*n*) for each experimental group/condition, given as a discrete number and unit of measurement
☐	☒	A statement on whether measurements were taken from distinct samples or whether the same sample was measured repeatedly
☐	☒	The statistical test(s) used AND whether they are one- or two-sided<br>*Only common tests should be described solely by name; describe more complex techniques in the Methods section.*
☐	☒	A description of all covariates tested
☐	☒	A description of any assumptions or corrections, such as tests of normality and adjustment for multiple comparisons
☐	☒	A full description of the statistical parameters including central tendency (e.g. means) or other basic estimates (e.g. regression coefficient) AND variation (e.g. standard deviation) or associated estimates of uncertainty (e.g. confidence intervals)
☐	☒	For null hypothesis testing, the test statistic (e.g. *F*, *t*, *r*) with confidence intervals, effect sizes, degrees of freedom and *P* value noted<br>*Give P values as exact values whenever suitable.*
☐	☒	For Bayesian analysis, information on the choice of priors and Markov chain Monte Carlo settings
☐	☒	For hierarchical and complex designs, identification of the appropriate level for tests and full reporting of outcomes
☐	☒	Estimates of effect sizes (e.g. Cohen's *d*, Pearson's *r*), indicating how they were calculated

Our web collection on statistics for biologists contains articles on many of the points above.

Software and code

Policy information about availability of computer code

Data collection	For data collection: PsychoPy (open source). Code for running experiment available online.
Data analysis	Costume-made code for analyzing the speech signal, in MATLAB. Codes available online.

For manuscripts utilizing custom algorithms or software that are central to the research but not yet described in published literature, software must be made available to editors and reviewers. We strongly encourage code deposition in a community repository (e.g. GitHub). See the Nature Portfolio guidelines for submitting code & software for further information.

Data

Policy information about availability of data

All manuscripts must include a data availability statement. This statement should provide the following information, where applicable:

- Accession codes, unique identifiers, or web links for publicly available datasets
- A description of any restrictions on data availability
- For clinical datasets or third party data, please ensure that the statement adheres to our policy

As the data set contains individuals' voices, ethical restrictions with regards to sharing the raw data online or on a public server applies. PLVs and the spectral content extracted from the speech signals to reconstruct Figure 2, and numerical data to reconstruct Figure 2 and Supplementary Figures are however publicly available at https://doi.org/10.17605/OSF.IO/DWRZ7 .

Human research participants

Policy information about studies involving human research participants and Sex and Gender in Research.

Reporting on sex and gender	Sex was determined based on self-reporting. We tested for and found sex differences in the first tested cohort (female=42, male =19) When collecting data from the second cohort, we aimed for a more balanced study sample (female=28, male=32). In this case, no sex differences were observed. Lastly, we also combined the data from both cohorts, stratified by sex and show that our sex differences are unlikely to drive our results.
Population characteristics	We collected information about age, sex, years of education, years of musical training and level of musical expertise. The sample consisted of young, healthy adults.
Recruitment	Participants were recruited from the near-by area and the university campus, via posters and online. Many of the participants are thus university students.
Ethics oversight	The Norwegian Center for Research Data

Note that full information on the approval of the study protocol must also be provided in the manuscript.

Field-specific reporting

Please select the one below that is the best fit for your research. If you are not sure, read the appropriate sections before making your selection.

☐ Life sciences ☒ Behavioural & social sciences ☐ Ecological, evolutionary & environmental sciences

For a reference copy of the document with all sections, see nature.com/documents/nr-reporting-summary-flat.pdf

Behavioural & social sciences study design

All studies must disclose on these points even when the disclosure is negative.

Study description	Quantitative data, in the form of speech recordings and questionnaires, was collected.
Research sample	Participants were recruited from the near-by area and the university campus, via posters and online. Many of the participants are university students (cohort 1: N=61, mean age=24 years, females = 42; cohort 2: N=60, mean age=30.5, females=28). We consider the sample to be representative of the adult Norwegian population.
Sampling strategy	A sampling size similar to the ones reported in the studies we are replicating, as well as suggested in the study protocol, was aimed for. A sample of around 60 participants should, based on these previous estimates, be sufficient for obtaining a bimodal distribution if one is exists in the Norwegian population. No sample-size calculation was performed. The sample was recruited based on convenience (people living in the near-by area to the university).
Data collection	Data was collected as 1) a 1-min speech recording, and 2) a questionnaire. A computer and over-ear headset was used for the speech recording, and the questionnaire was filled out on paper. No one but the participant and researcher was present during the experiment, and the researcher was not blind to the study hypothesis. Some of the participants in cohort 1 additionally took part in a short EEG study prior to the SSS test and filling our the questionnaire.
Timing	Data was collected between October 2021 and September 2023.
Data exclusions	Exclusion was based on the study protocol, namely if participants were silent for longer periods (4 s or more), spoke loudly instead of whispering, or because of extensive background noise. 11 participants were excluded.
Non-participation	No participant dropped out or declined participation.
Randomization	Participants were not allocated into experimental groups.

Reporting for specific materials, systems and methods

We require information from authors about some types of materials, experimental systems and methods used in many studies. Here, indicate whether each material, system or method listed is relevant to your study. If you are not sure if a list item applies to your research, read the appropriate section before selecting a response.

Materials & experimental systems

n/a	Involved in the study
☒	☐ Antibodies
☒	☐ Eukaryotic cell lines
☒	☐ Palaeontology and archaeology
☒	☐ Animals and other organisms
☒	☐ Clinical data
☒	☐ Dual use research of concern

Methods

n/a	Involved in the study
☒	☐ ChIP-seq
☒	☐ Flow cytometry
☒	☐ MRI-based neuroimaging
